# Psychological Health and Sleep Quality of Medical Graduates During the Second Wave of COVID-19 Pandemic in Post-epidemic Era

**DOI:** 10.3389/fpubh.2022.876298

**Published:** 2022-04-07

**Authors:** Honglin Wu, Huiyan Li, Xixi Li, Weijie Su, Hongxin Tang, Jia Yang, Zhong Deng, Lihua Xiao, Lixuan Yang

**Affiliations:** ^1^Department of Neurosurgery, The First Affiliated Hospital of Sun Yat-sen University, Guangzhou, China; ^2^Education Department, The First Affiliated Hospital of Sun Yat-sen University, Guangzhou, China

**Keywords:** anxiety, depression, sleep, medical graduates, vaccination status, COVID-19

## Abstract

Recently, a COVID-19 virus variant spread rapidly in Guangzhou, China, causing public panic. This study aimed to understand the psychological and sleep-related consequences of the secondary outbreak of the pandemic on medical students. In this cross-sectional survey-based study, participants anonymously completed structured questionnaires online from June 8–22, 2021. We collected participants' demographic and general information. Anxiety, depression, and sleep quality were measured using the Zung Self-Rating Anxiety Scale (SAS), Self-Rating Depression Scale (SDS), and Pittsburgh Sleep Quality Index (PSQI), respectively. Protective factors were assessed using the Coping Style Questionnaire (CSQ). Uni- and multivariate logistic regression analyses were performed examining factors associated with mental health and sleep quality problems. During the second wave of the pandemic in local outbreak areas in Guangzhou, China, more than one-third of medical students' mental health and sleep quality were affected. The prevalence of anxiety, depression, and poor sleep quality were 27.54%, 27.58%, and 18.19%, respectively. Students belonging to the Class of 2019, aged over 29 years, those with siblings, and those whose hometowns were in other provinces were more prone to the three health problems. Factors associated with an increased risk of mental health problems were vaccination status (adjusted odds ratio 1.603–1.839) and diet status (adjusted odds ratio 1.62–1.929). Positive coping styles served as protective factors (*p* < 0.05). We discovered that completed vaccination status, good diet, and positive coping styles were related to good mental health and sleep quality.

## Introduction

In December 2019, novel pneumonia caused by coronavirus disease 2019 (COVID-19) was first reported in Wuhan, Hubei Province, China (1). After implementing strict prevention and treatment protocols of COVID-19, on February 29, 2020, twenty provinces of mainland China announced no domestic COVID-19 cases (2). However, outside China, confirmed cases continued to increase worldwide. As a result, on March 15, 2020, the World Health Organization (WHO) declared the COVID-19 outbreak as a global pandemic (3). With the reversal of the epidemic at home and abroad, China was faced intense pressure due to imported cases (4).

The most severe localized outbreak caused by an imported case occurred in Guangzhou, China. The identified people shared the same RNA sequence as the highly infectious Delta variant strain (B.1.617.2), which was first found in India (5). Transmission associated with this outbreak in Guangdong was faster and more severe than the previous one. To respond to the unprecedented threat, Guangdong Province took a series of rigorous intervention measures, including mass testing, active case finding, community spread management, travel restrictions, and affected area lockdown to contain the spread (6).

The COVID-19 outbreak has substantially negatively impacted people's mental and physical well-being (7–9). Previous studies have shown that during the initial COVID-19 outbreak in China, more than half of the patients endured psychological problems such as anxiety and depression During the post-COVID-19 era, people experienced anxiety and depression accompanied by severe sleep problems (10–14). Several studies have reported a higher prevalence of poor sleep quality and clinical insomnia among healthcare workers worldwide involved in the outbreak control and COVID-19 patient care, compared to other groups of people, as they had to work long hours under intense pressure (15–19). Approximately one-third of medical staff in China endured anxiety, depression, and stress during the early phase of COVID-19(19). Because medical graduate students belong to the medical community, student depression and anxiety soared during the pandemic (20).

Although there have been many empirical studies on the mental health of medical students in China, few studies have surveyed anxiety symptoms, depression symptoms, and poor sleep quality of medical students at Sun Yat-Sen University during the second wave of COVID-19. Therefore, this cross-sectional study aimed to understand the prevalence of anxiety and depression, mental health protective factors, and sleep quality of medical students in China during the second COVID-19 outbreak and to lay the groundwork for further measures for at-risk medical students.

## Materials and Methods

### Recruitment

An online cross-sectional survey was designed and conducted during the second wave of the COVID-19 pandemic in June 2021 at The First Affiliated Hospital of Sun Yat-Sen University. The university's institutional review board approved the study. Participants were medical students belonging to the institution. On June 8, 2021, schools and hospitals issued a stay-at-home order and took strict measures to ensure that students stayed within the campus and did not have to leave. This led to experimental and clinical learning interruptions. During this period, we announced our study's survey to the entire population of over 1,400 medical graduate students at The First Affiliated Hospital of Sun Yat-Sen University through text messages. It was published using the Wenjuanxing platform (https://www.wjx.cn/app/survey.aspx) on June 23. Data collection continued until survey submission ceased three consecutive days (June 25, 2021).

### Survey Design

The survey comprised single-choice questions and free-text response fields for elaboration. It consisted of the following five sections:

#### Demographics

This section included questions related to participants' age, gender, program classification (master's or doctoral), hometown location, family structure, diet over the last 2 weeks, and vaccination (BBIBP-CorV or Sinovac Coronavac) status. At present, being the single-child account for a certain proportion of Chinese college students. Most of them grow up spoiled and do not experience too many difficulties, so they may have different psychological states when they are quarantined during the epidemic. We think this is a meaningful factor. During the epidemic, many students eat takeout. Compared with the school canteen, the food for takeout is unreasonable and not very hygienic. Irregular and unhealthy takeout diets cause harm to the physical and mental health of students. So we also think “time of eating in canteen or takeout” is a significant factor.

#### Anxiety Symptoms

Anxiety was assessed over 2 weeks using the Zung Self-Rating Anxiety Scale (SAS) developed by Zung (21, 22). It consists of 20 items. Responses are rated on a four-point scale, ranging from 1(no or very little time) to 2(sometimes), to 3(good part of the time), and to 4(most or all of the time). The raw score is the sum of all the responses. The standard score is calculated as an integer value 1.25 times the raw score. The current study used an index score cutoff of ≥50 to diagnose anxiety. The SAS has been shown to have good internal consistency with a Cronbach's alpha of 0.82(23). The Chinese translation of SAS has been previously validated (24).

#### Depressive Symptoms

The Self-Rating Depression Scale (SDS) is a 20-question self-report survey aimed to assess a patient's level of depression over the past 2 weeks (25). Participants described how frequently they experienced each symptom on a 4-point scale: 'a little of the time', 'some of the time', 'good part of the time', or 'most of the time'. The frequency was converted into an integer between 1 and 4. The scores of each item in the 20 items were added together to obtain the total rough score. Then multiply the rough total score by 1.25 and take the integer part to get the standard score. We used an SDS score of 50 as the cut-off point for clinical significance (21). SDS is suitable for discriminating between depressed and non-depressed participants (26). Previous research showed that the Cronbach's alpha coefficients of SDS was 0.81 (23).

#### Sleep Quality

Sleep quality was evaluated using the Pittsburgh Sleep Quality Index (PSQI) (27), a self-report scale that assesses the sleep quality of the respondents over 2 weeks. The PQSI was mainly composed of 18 items, with a total of 7 components including sleep quality, sleep onset time, sleep time, sleep efficiency, sleep disturbance, hypnotic drugs and daytime dysfunction. Each component was scored on a 0–3 scale. The sum of the scores of the 7 components was the total score of the PSQI. The higher the score, the worse the sleep quality (28). The total score ranges from 0 to 21, and a score of >7 is indicative of poor sleep quality in this study. Previous study showed that the Cronbach's alpha coefficients of the seven main components and each item of PQSI were 0.8420 and 0.8519, respectively among Chinese people (29).

#### Coping Style Questionnaire

The Coping Style Questionnaire (CSQ) was developed by Xie (30). It is based on the Ways of Coping questionnaire by Folkman and Lazarus (31). The CSQ is a 20-item self-report that includes two dimensions, active coping (12-item) and passive coping (8-item). Participants rated the frequency with which they adopted the strategy in the face of stress on a four-point scale, ranging from 0 (never) to 3 (very often). The final score is the standard score (z-score) of active coping (12-item) minus the standard score of passive coping (8-item), and a score of >0 is indicative of positive coping style in this study. Xie validated the CSQ in a Chinese sample aged 20–65 years. The instrument has been commonly used in China and the internal consistency measured by Cronbach's alpha was reported to be 0.78 (32).

### Statistical Analysis

All analyses were conducted using Statistical Package for the Social Sciences (SPSS) Version 19.0.0 (SPSS Inc., Chicago, IL, USA). Descriptive analyses were used to estimate means (M), standard deviations (SD), and prevalence of mental health problems. The data were analyzed using chi-square tests (χ^2^) to compare differences between groups, and univariate and multivariate logistic regression analyses were performed to examine factors associated with mental health and sleep quality problems. The associations were presented using odds ratios (ORs) and their 95% confidence intervals (CIs) in the unadjusted analyses and adjusted ORs (AORs) and their 95% CIs in the adjusted analysis. *P*-values were two-sided and considered significant at *P* < 0.05, or *P* < 0.001.

## Results

Table 1 presents the participants' general characteristics. Of the 1,336 participants, 636 (47.60%) were men, and 700 (52.40%) were women. Among these participants, 876 (65.57%) were graduate students, 460 (34.43%) were doctoral students. A total of 633 (47.38%) medical students chose a negative coping style. Other detailed sample characteristics, including age, hometown location, family structure, diet for the past 2 weeks, and vaccination status, are presented below.

**Table 1:** Demographic characteristics of study sample (*N* = 1,336).

**Variable**	**No**	**(%)**
**Gender**
Male	636	47.60%
Female	700	52.40%
**Grade**
Class of 2020	597	44.69%
Class of 2019	428	32.04%
Class of 2018	311	23.28%
**Classification**
Master's	876	65.57%
Doctera	460	34.43%
**Age(years)**
20–23	69	5.16%
24–26	707	52.92%
27–29	372	27.84%
Over 29	188	14.07%
**Hometown**
This province	430	32.19%
Other provinces	906	67.81%
**Family structure**
Without siblings	464	34.73%
With siblings	872	65.27%
**Diet in 2 weeks**		
Canteen	380	28.44%
Half of canteen	483	36.15%
Takeout	473	35.40%
Vaccination Status		
0 dose	180	13.47%
1 dose	528	39.52%
2 doses	628	47.01%
**Coping style**		
Negative	633	47.38%
Positive coping	703	52.62%

**Figure 1:**
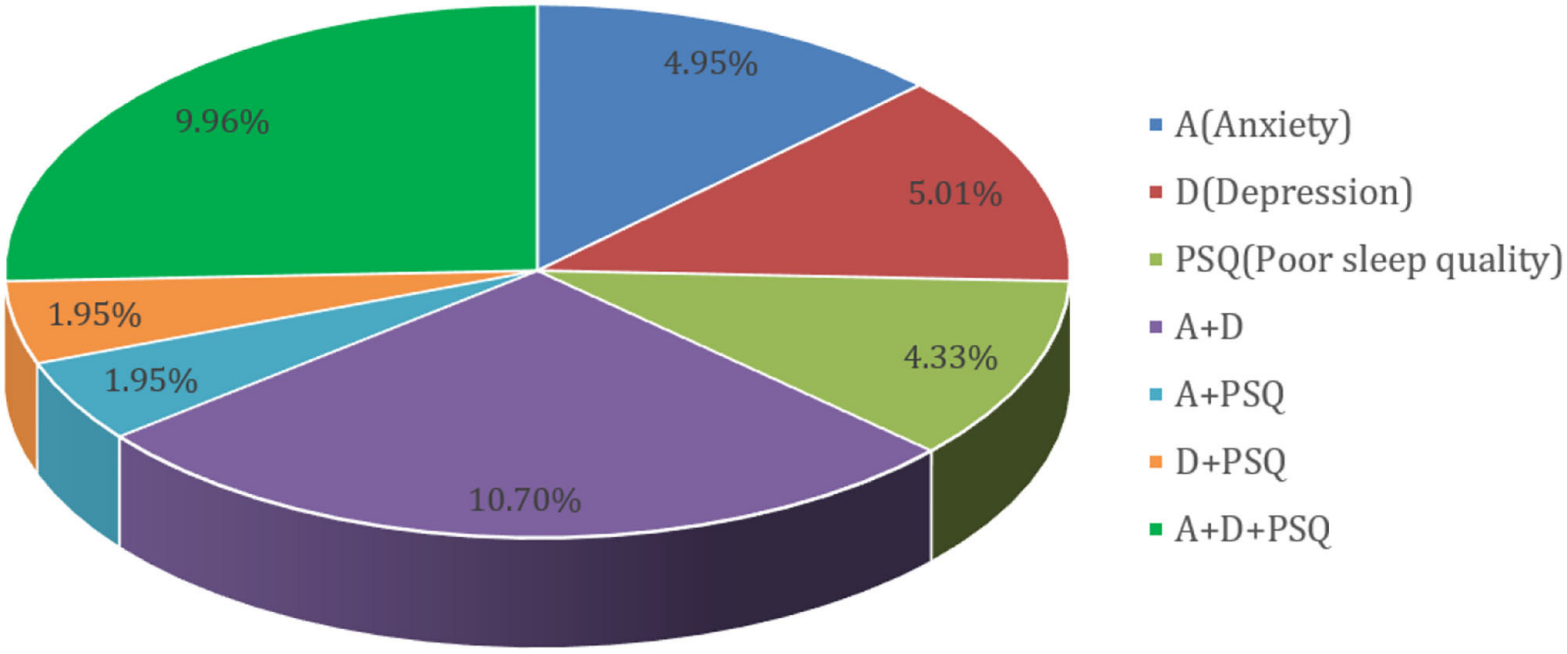
Prevalence rate of probable anxiety symptoms, depressive symptoms and poor sleep quality (*N* = 1,026).

Figure 1 shows that among the 1,336 participants, 38.85% had potential anxiety symptoms, depressive symptoms, and poor sleep quality. The prevalence of probable anxiety symptoms, depressive symptoms, and poor sleep quality were 27.54%, 27.58%, and 18.19%, respectively. Anxiety and depressive symptoms were present in 10.07% of the participants, while all three health problems occurred in 9.96% of all participants. Poor sleep quality and anxiety symptoms, poor sleep quality, and depression symptoms, represented 1.95% of the total.

In the univariate logistic regression model (Table 2), grade, age (years), family structure, and coping style were significantly associated with the three health problems. Anxiety symptoms were markedly found in the students belonging to the Class of 2019 (*p* < 0.01), aged over 29 years (*p* < 0.01), and living in the this province *p* < 0.01), with a significant between-group difference. The prevalence of anxiety symptoms was significantly higher among students from families with multiple children (*p* < 0.05). Hometown location (OR = 1.508, 95% CI: 1.173–1.938) and diet for the past 2 weeks (OR = 1.603, 95% CI: 1.122–2.291) were associated with an increased risk of anxiety symptoms. Compared with students in other grades, students in the Class of 2019 reported the highest rate of depression symptoms and poor sleep quality. Students older than 29 and having siblings also had more depressive symptoms and poor sleep quality. Graduates who ate half the time in the canteen were 1.62 times more likely to have depression symptoms than students who ate in the canteen all the time. Those who were not vaccinated were 1.603–1.779 times more likely to have anxiety or depressive symptoms than individuals who had received two vaccine doses. Similarly, hometown was related to medical students' poor sleep quality during the local COVID-19 outbreak. However, gender, classification, diet for the last 2 weeks, and vaccination status were not associated with poor sleep quality (*p* > 0.05).

**Table 2:** Factors associated with probable anxiety, depression and sleep quality problem using univariate logistic regression analyses among 1,336 Chinese medical graduates.

**Variable**	**Anxiety**	**Depression**	**Poor sleep quality**
	**OR (95% CI)**	**OR (95% CI)**	**OR (95% CI)**
**Gender**			
Male	1.212(0.953–1.542)	0.946(0.744–1.204)	0.901(0.722–1.124)
Female	1	1	1
**Grade**			
Class of 2020	1.659(1.191–2.31)^***^	1.599(1.149–2.224)^**^	1.428(1.064–1.915)^**^
Class of 2019	1.888(1.336–2.67)^***^	1.891(1.34–2.668)^***^	1.943(1.428–2.646)^***^
Class of 2018	1	1	1
**Classification**			
Master's	1.064(0.825–1.371)	1.184(0.917–1.529)	0.842(0.668–1.06)
Doctorate	1	1	1
**Age (years)**			
20-23	0.704(0.387–1.281)	1.023(0.566–1.851)	0.484(0.276–0.849)^**^
24-26	0.638(0.454–0.897)^**^	0.911(0.644–1.291)	0.398(0.286–0.552)^***^
27-29	0.534(0.365–0.782)^***^	0.599(0.404–0.889)^**^	0.342(0.238–0.491)^***^
Over 29	1	1	1
**Hometown**			
This province	1.508(1.173–1.938)^***^	1.15(0.892–1.483)	1.275(1.009–1.611)^**^
Other provinces	1	1	1
**Family structure**			
Without siblings	0.63(0.484–0.82)^***^	0.71(0.548–0.921)^**^	0.587(0.462–0.745)^***^
With siblings	1	1	1
**Diet in 2 weeks**			
Eating in canteen	0.842(0.607–1.169)	0.684(0.493–0.95)^**^	0.826(0.624–1.094)
Half of in canteen	1.92(1.449–2.545)^***^	1.62(1.228–2.138)^**^	1.091(0.842–1.414)
Takeout	1	1	1
**Vaccination Status**			
0 dose	1.603(1.122–2.291)^**^	1.779(1.251–2.529)^***^	1.083(0.774–1.516)
1 dose	1.222(0.941–1.588)	1.136(0.873–1.477)	0.793(0.624–1.008)
2 doses	1	1	1
**Coping style**			
Negative style	4.348(3.342–5.658)^***^	6.542(4.942–8.66)^***^	1.501(1.203–1.873)^***^
Positive style	1	1	1

** *p < 0.05*

*** *p < 0.001*.

Multivariate logistic regression (Table 3) showed that grade (AOR = 1.617–2.147), age (AOR = 0.261–0.591), coping style (AOR = 1.453–6.419), and diet for the past 2 weeks were significantly associated with an increased risk of the three health problems. The classification was a risk factor for anxiety but belonging to a single-child family was a non-risk factor for depression. Individuals' full vaccination status had a protective effect on their mental health. Similarly, a diet based on eating take-out food was a risk factor for poor sleep quality. Graduate students in the Class of 2019 (AOR = 2.177), aged over 29, whose hometown was in the same province, who belonged to a single-child family, and who had a diet based on eating take-out food were more likely to report poor sleep quality.

**Table 3:** Factors associated with probable anxiety, depression and sleep quality problem using multivariate logistic regression analyses among 1,336 Chinese medical graduates.

**Variable**	**Anxiety**	**Depression**	**Poor sleep quality**
	**AOR (95% CI)**	**AOR (95% CI)**	**AOR (95% CI)**
**Gender**			
Male	1.233(0.937–1.623)	0.922(0.697–1.218)	0.856(0.672–1.09)
Female	1	1	1
**Grade**			
Class of 2020	2.147(1.454–3.17)^***^	1.617(1.098–2.382)^**^	1.626(1.171–2.257)^**^
Class of 2019	2.17(1.465–3.215)^***^	1.801(1.218–2.665)^**^	2.177(1.563–3.032)^***^
Class of 2018	1	1	1
**Classification**			
Master's	1.556(1.013–2.391)^**^	0.955(0.617–1.477)	1.21(0.829–1.767)
Doctorate	1	1	1
**Age (years)**			
20–23	0.345(0.156–0.766)^**^	0.782(0.35–1.745)	0.307(0.151–0.624)^***^
24–26	0.33(0.191–0.568)^***^	0.714(0.412–1.239)	0.261(0.16–0.427)^***^
27–29	0.504(0.324–0.784)^**^	0.591(0.373–0.936)^**^	0.31(0.209–0.46)^***^
Over 29	1	1	1
**Hometown**			
This province	1.553(1.168–2.063)^***^	1.041(0.777–1.395)	1.312(1.021–1.685)^**^
Other provinces	1	1	1
**Family structure**			
Without siblings	0.738(0.548–0.992)^**^	0.802(0.595–1.082)	0.708(0.547–0.916)^**^
With siblings	1	1	1
**Diet in 2 weeks**			
Eating in canteen	0.889(0.622–1.271)	0.761(0.529–1.095)	0.683(0.505–0.924)^**^
Half of in canteen	1.929(1.419–2.624)^***^	1.658(1.217–2.258)^***^	0.963(0.733–1.265)
Takeout	1	1	1
**Vaccination Status**			
0 dose	1.599(1.076-2.376)^**^	1.839(1.237–2.73)6^**^	0.986(0.692–1.403)
1 dose	1.366(1.024–1.821)^**^	1.2140.9061.627	0.793(0.617–1.02)
2 doses	1	1	1
**Coping style**			
Negative style	4.277(3.247–5.633)^***^	6.419(4.808–8.569)^***^	1.453(1.151–1.834)^**^
Positive style	1	1	1

** *p < 0.05*

*** *p < 0.001*.

## Discussion

To the best of our knowledge, this study was the first to investigate the psychological and sleep consequences of the second wave of the COVID-19 pandemic and its influencing factors on medical graduate students from a large general hospital cohort, approximately 2 weeks into the implementation of restrictive government rules, and stringent school, and hospital measures.

Our study has several significant findings. First, mental health and sleep quality problems were prevalent in approximately 38.85% of the participants during the second wave of the COVID-19 epidemic. The prevalence of probable anxiety symptoms, depressive symptoms, and poor sleep quality were 27.54%, 27.58%, and 18.19%, respectively. Second, graduate students in the Class of 2019, older than 29, and residing in this province had a higher prevalence of anxiety, depression, and poor sleep quality symptoms than other students. Third, the prevalence of anxiety symptoms was significantly higher among students with siblings. Graduate students born in non-single-child families also had a higher rate of depressive symptoms and poor sleep quality. There were no significant differences in the prevalence of anxiety symptoms, depressive symptoms, and poor sleep quality according to gender or program classification. Third, positive coping and having siblings were protective factors for anxiety, depression, and poor sleep symptoms in all students. Those whose hometowns were in different provinces and whose diet consisted of eating canteen food only half of the time had an increased risk of probable anxiety symptoms. Fourth, individuals who were not vaccinated were 1.603–1.779 times more likely than individuals who received two shots of a vaccine to have anxiety or depressive symptoms. However, sex, classification, diet over the preceding 2 weeks, and vaccination status were not associated with poor sleep quality. Fifth, multiple factors such as grade, age, program classification, hometown, coping style, and diet over the preceding 2 weeks were also associated with an increased likelihood of all three health problems. Moreover, individuals' completed vaccination status had a protective effect on mental health. Therefore, our study findings could frame appropriate psychological interventions to prevent the occurrence of mental health and sleep problems.

In our study, we used a standardized questionnaire to assess mental health and sleep problems during the second outbreak of the COVID-19 epidemic in China. A previous study showed that 43.77% of Chinese college students had depressive symptoms, and 20.60% had anxiety symptoms during COVID-19(33). Based on a meta-analysis, the prevalence of anxiety ranged from 8.54–88.30%, with a mean of 27.22%, and depression ranged from 13.10%−76.21% with a mean of 32.74%, among 35,160 Chinese medical students (34). A previous study found that 39% of graduate (law and medical school) students in the United States had anxiety, and 32% had depression. However, our survey showed that over 27% of Chinese graduate students had anxiety or depression symptoms. The incidence of both symptoms was similar. The lower incidence of anxiety symptoms may be due to medical students' ability to better adapt to the epidemic and their experience of the first wave of COVID-19.

Our survey revealed no significant differences between men and women in terms of anxiety symptoms, depression symptoms, and sleep disturbances, contrary to other studies (35, 36). However, we found that students in the Class of 2019 (second grade) had the highest risk of mental problems. This increased risk may be due to the students' delayed academic progress and difficulty coping with clinical practice and research as they did not get the opportunity to go to the laboratory or partake in clinical practice because of the sudden onset of the pandemic (37).

A previous study showed that age was negatively correlated with anxiety and depression. However, our study found that graduate students aged above 29 years, had a higher proportion of mental problems during the second wave of COVID-19(38). A possible reason for this finding may be that they face pressure to support their families (39).

We found that students whose hometowns belong to the same province had a higher prevalence of anxiety symptoms than those whose hometowns were in other provinces. This elevated risk may be because the students in this province worry more about their families' risk of contracting the COVID-19 infection.

Our study's findings suggested that 48.3% of students in the Class of 2019 had sleep problems, significantly higher than those from other classes. In addition, more than half of the students aged over 29 years had poor sleep quality. This risk may be due to the increased stress from the ongoing COVID-19 pandemic and academic expectations (40, 41).

It has been reported in the literature that diet and sleep have complex interactions (15). In our study, a good and regular diet, such as eating in the canteen, is a protective factor for sleep. In addition, studies have shown that before and during the COVID-19 emergency, an increase in sleeping hours, sleep latency, and wake-up time impaired sleep quality and exacerbated insomnia symptoms.

We also found that a positive coping style may ensure good sleep and mental health. These involved positive appraisal and thinking, distancing, problem-solving, and help-seeking behaviors. These active coping strategies can enhance mental health by promoting an individual's sense of control over a chaotic environment and creating opportunities to establish satisfying relationships with a support network (42, 43). An increasing amount of evidence suggests that positive coping is a protective factor for mental health. For example, it has been reported that the using a positive coping style could promote university students' academic adjustment and reduce displays of maladaptive behaviors (44). Negative coping techniques were positively related to psychological distress during the COVID-19 outbreak (45). Previous studies have shown that higher negative coping technique scores were related to increased prevalence of anxiety symptoms. In contrast, greater use of positive coping techniques was related to a reduced prevalence of anxiety symptoms. The association between negative coping styles and anxiety symptoms is mediated by sleep quality (46). Our research indicates that positive coping has a powerful protective effect against anxiety and depression symptoms. By adopting proper coping methods and equipping oneself with the necessary coping and stress management skills, associated high levels of perceived stress and anxiety could be mitigated (47).

We found that vaccination status potentially influenced mental health. Unvaccinated students had a high incidence of anxiety and depression symptoms during the second wave of COVID-19. Despite the good safety and efficacy of COVID-19 vaccines made in China, some public skepticism about the vaccines persists (48). They may be concerned about the uncertainty of its effectiveness, side effects, and effective duration (49). It should be noted that mass exposure to media coverage of the COVID-19 or vaccine was associated with an increased risk of probable acute stress and anxiety symptoms (50). Our findings are consistent with COVID-19 related anxiety and fear of infection. Health-related consequences correlated significantly positively with vaccine acceptance (51). In addition, people who refuse the vaccine may pay more attention to daily self-protection. However, a study indicated that very high adherence to personal hygiene factors (such as hand washing, use of disinfectants, wiping down knobs, and surfaces, etc.) predicted f higher anxiety levels (52).

Further, we found that eating at the canteen was more protective from anxiety and depression than eating take-away food. Increasing evidence indicates a strong association between a poor diet and the exacerbation of mood disorders, including anxiety and depression (53). Take-out foods tend to be unhealthy because they are more energy-dense and nutrient-poor than canteen or home-cooked foods. They often contain high quantities of fat, salt, and sugar contents, associated with weight gain and various adverse health outcomes (54). In China, many hot set meals on take-out platforms have the problem of excess energy supply (55). Improving diet quality and subsequent gut health may benefit individuals' mental health (56). Good diet and proper nutrition play essential roles in psychological and physical well-being (57).

This study has several limitations. First, although our sample was large and participants had good educational backgrounds, all the students were initially sampled from one university in Guangzhou, Guangdong province. Hence, it is uncertain whether our findings could be generalized to all students, especially graduate students who lived in local outbreak regions. Second, the duration of the study was short. As the second wave of COVID-19 was short-lived, we only focused on the symptoms that developed during the 2 weeks. Third, no causality could be made between COVID-19 and mental health problems due to the cross-sectional design of our study. Finally, although the measurements used in the current study have good psychometric properties, they are self-reports for screening rather than clinical diagnoses.

## Conclusions

This large-scale survey of medical students in China demonstrated that about 38.85% of students had probable depression, anxiety, or poor sleep quality symptoms during the second wave of the COVID-19 epidemic. In addition, multiple factors, such as being unvaccinated, negative coping style, unhealthy diet, grade, age over 29 years, having siblings, having a hometown in the same province, and classification were associated with an increased risk of mental health and sleep problems. These findings may have important implications for prevention, psychosocial interventions, and future research. Notably, mental health services should be made available for graduate students.

## Data Availability Statement

The original contributions presented in the study are included in the article/supplementary material, further inquiries can be directed to the corresponding author/s.

## Ethics Statement

The studies involving human participants were reviewed and approved by Ethics Committee the First Affiliated Hospital of Sun Yat-sen University (number: [2021]771). The patients/participants provided their written informed consent to participate in this study.

## Author Contributions

HL and LX conceived and designed the questionnaire. HL recruitment and payment of participants. XL, WS, HT, ZD, and JY analyzed the data. LY and HW wrote and revised the paper. All the authors have approved the manuscript and agreed with submission to your esteemed journal.

## Funding

This work was supported by the National Social Science Fund of China (Grant no. 82073049), and the Scientific and Technological Planning Project of Guangzhou City (Grant no. 201903010093).

## Conflict of Interest

The authors declare that the research was conducted in the absence of any commercial or financial relationships that could be construed as a potential conflict of interest.

## Publisher's Note

All claims expressed in this article are solely those of the authors and do not necessarily represent those of their affiliated organizations, or those of the publisher, the editors and the reviewers. Any product that may be evaluated in this article, or claim that may be made by its manufacturer, is not guaranteed or endorsed by the publisher.

## References

[1] Hui DS EI A Madani TA Ntoumi F Kock R Dar O. The continuing 2019-Ncov epidemic threat of novel coronaviruses to global health - the latest 2019 novel coronavirus outbreak in Wuhan, China. Int J Infect Dis. (2020) 91:264–6. 10.1016/j.ijid.2020.01.009 31953166 PMC7128332

[2] China Daily in March 28 2020. Available online at: https://www.chinadaily.com.cn/a/202003/28/WS5e7f2ba6a310128217282b8c.html (accessed March 28, 2020).

[3] Coronavirus Outbreak is a Pandemic Says WHO. Available online at: https://www.who.int/zh/director-general/speeches/detail/who-director-general-s-opening-remarks-at-the-media-briefing-on-covid-19—11-march-2020 (accessed March 11, 2020).

[4] Zhu J Zhang Q Jia C Xu S Lei J Chen J. Challenges caused by imported cases abroad for the prevention and control of covid-19 in China. Front. Med. (2021) 8:573726. 10.3389/fmed.2021.573726 34095156 PMC8172980

[5] Adam D. What scientists know about new, fast-spreading coronavirus variants. Nature. (2021) 594:19–20. 10.1038/d41586-021-01390-4 34031583

[6] Zhang M Xiao J Deng A Zhang Y Zhuang Y Hu T. Transmission dynamics of an outbreak of the covid-19 delta variant B.1.617.2 - Guangdong province, China, may-june 2021. China CDC weekly. (2021) 3:584–6. 10.46234/ccdcw2021.151 34594941 PMC8392962

[7] Bao Y Sun Y Meng S Shi J Lu L. 2019-ncov epidemic: address mental health care to empower society. Lancet (London, England). (2020) 395:e37–8. 10.1016/S0140-6736(20)30309-3 32043982 PMC7133594

[8] Kelly BD. Coronavirus disease: challenges for psychiatry. Br J Psychiatry. (2020) 217:352–3. 10.1192/bjp.2020.86 32293555 PMC7205546

[9] Xiang YT Jin Y Cheung T. Joint international collaboration to combat mental health challenges during the coronavirus disease 2019 pandemic. JAMA psychiatry. (2020) 77:989–90. 10.1001/jamapsychiatry.2020.1057 32275289

[10] Wang C Pan R Wan X Tan Y Xu L Ho CS. Immediate psychological responses and associated factors during the initial stage of the 2019 coronavirus disease (covid-19) epidemic among the general population in China. Int J Environ Res. (2020) 17. 10.3390/ijerph17051729 PMC7084952 32155789

[11] Rossi R Socci V Talevi D Mensi S Niolu C Pacitti F. Covid-19 pandemic and lockdown measures impact on mental health among the general population in Italy. Front Psychiatry. (2020) 11:790. 10.3389/fpsyt.2020.00790 32848952 PMC7426501

[12] Xie J Li X Luo H He L Bai Y Zheng F. Depressive symptoms, sleep quality and diet during the 2019 novel coronavirus epidemic in China: a survey of medical students. Public Health Front. (2020) 8:588578. 10.3389/fpubh.2020.588578 33575239 PMC7870982

[13] Pappa S Ntella V Giannakas T Giannakoulis VG Papoutsi E Katsaounou P. Prevalence of depression, anxiety, and insomnia among healthcare workers during the covid-19 pandemic: a systematic review and meta-analysis. Brain Behav Immun. (2020) 88:901–7. 10.1016/j.bbi.2020.05.026 32437915 PMC7206431

[14] Alvaro PK Roberts RM Harris JK. A Systematic review assessing bidirectionality between sleep disturbances, anxiety, and depression. Sleep. (2013) 36:1059–68. 10.5665/sleep.2810 23814343 PMC3669059

[15] Marelli S Castelnuovo A Somma A Castronovo V Mombelli S Bottoni D. Impact of covid-19 lockdown on sleep quality in university students and administration staff. J Neurol. (2021) 268:8–15. 10.1007/s00415-020-10056-6 32654065 PMC7353829

[16] Gualano MR Lo Moro G Voglino G Bert F Siliquini R. Effects of covid-19 lockdown on mental health and sleep disturbances in Italy. Int. J. Environ. Res. (2020) 17. 10.3390/ijerph17134779 PMC7369943 32630821

[17] Killgore WDS Cloonan SA Taylor EC Fernandez F Grandner MA Dailey NS. Suicidal ideation during the covid-19 pandemic: the role of insomnia. Psychiatry Res. (2020) 290:113134. 10.1016/j.psychres.2020.113134 32505030 PMC7255187

[18] Zhou SJ Wang LL Yang R Yang XJ Zhang LG Guo ZC. Sleep problems among Chinese adolescents and young adults during the coronavirus-2019 pandemic. Sleep Med. (2020) 74:39–47. 10.1016/j.sleep.2020.06.001 32836185 PMC7274988

[19] Dong F Liu HL Yang M Lu CL Dai N Zhang Y. Immediate psychosocial impact on healthcare workers during covid-19 pandemic in China: a systematic review and meta-analysis. Front Psychol. (2021) 12:645460. 10.3389/fpsyg.2021.645460 34122233 PMC8192844

[20] Woolston C. Signs of depression and anxiety soar among Us graduate students during pandemic. Nature. (2020) 585:147–8. 10.1038/d41586-020-02439-6 32811983

[21] Zung WW. A Self-Rating depression scale. Arch Gen Psychiatry. (1965) 12:63–70. 10.1001/archpsyc.1965.01720310065008 14221692

[22] Zung WW. A Rating instrument for anxiety disorders. Psychosomatics. (1971) 12:371–9. 10.1016/S0033-3182(71)71479-0 5172928

[23] Tanaka-Matsumi J Kameoka VA. Reliabilities and concurrent validities of popular self-report measures of depression, anxiety, and social desirability. J Consult Clin Psychol. (1986) 54:328–33. 10.1037/0022-006X.54.3.328 3722561

[24] Ramirez SZ Lukenbill J. Psychometric properties of the zung self-rating anxiety scale for adults with intellectual disabilities (Sas-Id). J Dev Phys Disabil. (2008) 20:573–80. 10.1007/s10882-008-9120-x

[25] Pang Z Tu D Cai Y. Psychometric properties of the Sas, Bai, and S-Ai in Chinese university students. Front Psychol. (2019) 10:93. 10.3389/fpsyg.2019.00093 30766501 PMC6365890

[26] Dunstan DA Scott N. Clarification of the cut-off score for zung's self-rating depression scale. BMC Psychiatry. (2019) 19:177. 10.1186/s12888-019-2161-0 31185948 PMC6558728

[27] Buysse DJ Reynolds CF 3rd Monk TH Berman SR Kupfer DJ. The pittsburgh sleep quality index: a new instrument for psychiatric practice and research. Psychiatry Res. (1989) 28:193–213. 10.1016/0165-1781(89)90047-4 2748771

[28] Smith MT Wegener ST. Measures of sleep: the insomnia severity index, Medical Outcomes Study (Mos) sleep scale, Pittsburgh Sleep Diary (Psd), and Pittsburgh Sleep Quality Index (Psqi). Arthritis Rheumatol. (2003) 49:S184–96. 10.1002/art.11409

[29] Liu XC Tang MQ Hu L Wang A Hu H Zhao G Reliability and validity of pittsburgh sleep quality index. Chin. J. Psychiatry. (1996) 29 103–107.

[30] Xie Y. Reliability and validity of the simplified coping style questionnaire. Chinese Journal of Clinical Psychology. (1998) 6.

[31] Folkman S Lazarus RS. Coping as a mediator of emotion. J Pers Soc Psychol. (1988) 54:466–75. 10.1037/0022-3514.54.3.466 3361419

[32] Xie YN. Reliability and validity of the simplified coping style questionnaire. Chin J Clin Psychol. (1998) 6:114–5.30518288

[33] Zhan H Zheng C Zhang X Yang M Zhang L Jia X. Chinese college students' stress and anxiety levels under covid-19. Front Psychiatry. (2021) 12:615390. 10.3389/fpsyt.2021.615390 34177635 PMC8222572

[34] Mao Y Zhang N Liu J Zhu B He R Wang X. Systematic review of depression and anxiety in medical students in China. BMC Med Educ. (2019) 19:327. 10.1186/s12909-019-1744-2 31477124 PMC6721355

[35] Ma Z Zhao J Li Y Chen D Wang T Zhang Z. Mental health problems and correlates among 746 217 college students during the coronavirus disease 2019 outbreak in China. Epidemiol Psychiatr Sci. (2020) 29:e181. 10.1017/S2045796020000931 33185174 PMC7681173

[36] Zhang J Yang Z Wang X Li J Dong L Wang F. The relationship between resilience, anxiety and depression among patients with mild symptoms of covid-19 in China: a cross-sectional study. J Clin Nurs. (2020) 29:4020–9. 10.1111/jocn.15425 32702192 PMC7404600

[37] Yusoff MS Abdul Rahim AF Baba AA Ismail SB Mat Pa MN Esa AR. The impact of medical education on psychological health of students: a cohort study. Psychol Health Med. (2013) 18:420–30. 10.1080/13548506.2012.740162 23140393

[38] Christensen H Jorm AF Mackinnon AJ Korten AE Jacomb PA Henderson AS. Age differences in depression and anxiety symptoms: a structural equation modelling analysis of data from a general population sample. Psychol Med. (1999) 29:325–39. 10.1017/S0033291798008150 10218924

[39] Madewell ZJ Yang Y Longini IM Jr Halloran ME Dean NE. Household transmission of sars-cov-2: a systematic review and meta-analysis. JAMA Network Open. (2020) 3:e2031756. 10.1001/jamanetworkopen.2020.31756 33315116 PMC7737089

[40] Pizzonia KL Koscinski B Suhr JA Accorso C Allan DM Allan NP. Insomnia during the covid-19 pandemic: the role of depression and covid-19-related risk factors. Cogn Behav Ther. (2021) 50:246–60. 10.1080/16506073.2021.1879241 33787448 PMC8140992

[41] Peuhkuri K Sihvola N Korpela R. Diet promotes sleep duration and quality. Nutr Res. (2012) 32:309–19. 10.1016/j.nutres.2012.03.009 22652369

[42] Zhang C Ye M Fu Y Yang M Luo F Yuan J. The Psychological impact of the covid-19 pandemic on teenagers in China. J Adolesc Health. (2020) 67:747–55. 10.1016/j.jadohealth.2020.08.026 33041204 PMC7543885

[43] Compas BE Malcarne VL Fondacaro KM. Coping with stressful events in older children and young adolescents. J Consult Clin Psychol. (1988) 56:405–11. 10.1037/0022-006X.56.3.405 3397433

[44] Russell J Rosenthal D Thomson G. The international student experience: three styles of adaptation. High Educ. (2010) 60:235–49. 10.1007/s10734-009-9297-7

[45] Tada A. The Associations among psychological distress, coping Style, and health habits in japanese nursing students: a cross-sectional study. Int J Environ. Res. (2017) 14. 10.3390/ijerph14111434 PMC5708073 29165395

[46] Xiong W Liu H Gong P Wang Q Ren Z He M. Relationships of coping styles and sleep quality with anxiety symptoms among Chinese adolescents: a cross-sectional study. J Affect Disord. (2019) 257:108–15. 10.1016/j.jad.2019.07.032 31301610

[47] Garbóczy S Szemán-Nagy A Ahmad MS Harsányi S Ocsenás D Rekenyi V. Health anxiety, perceived stress, and coping styles in the shadow of the covid-19. BMC psychology. (2021) 9:53. 10.1186/s40359-021-00560-3 33823945 PMC8022303

[48] Lin Y Hu Z Zhao Q Alias H Danaee M Wong LP. Understanding covid-19 vaccine demand and hesitancy: a nationwide online survey in China. PLoS Negl Trop Dis. (2020) 14:e0008961. 10.1371/journal.pntd.0008961 33332359 PMC7775119

[49] Kwok KO Li KK Wei WI Tang A Wong SYS Lee SS. Editor's choice: influenza vaccine uptake, covid-19 vaccination intention and vaccine hesitancy among nurses: a survey. Int J Nurs Stud. (2021) 114:103854. 10.1016/j.ijnurstu.2020.103854 33326864 PMC7831770

[50] Neria Y Sullivan GM. Understanding the mental health effects of indirect exposure to mass trauma through the media. Jama. (2011) 306:1374–5. 10.1001/jama.2011.1358 21903818 PMC3637659

[51] Bendau A Plag J Petzold MB Ströhle A. Covid-19 vaccine hesitancy and related fears and anxiety. Int Immunopharmacol. (2021) 97:107724. 10.1016/j.intimp.2021.107724 33951558 PMC8078903

[52] Solomou I Constantinidou F. Prevalence and predictors of anxiety and depression symptoms during the covid-19 pandemic and compliance with precautionary measures: age and sex matter. Int J Environ. Res. (2020) 17. 10.3390/ijerph17144924 PMC7400373 32650522

[53] Adan RAH van der Beek EM Buitelaar JK Cryan JF Hebebrand J Higgs S. Nutritional psychiatry: towards improving mental health by what you eat. Eur Neuropsychopharmacol. (2019) 29:1321–32. 10.1016/j.euroneuro.2019.10.011 31735529

[54] Janssen HG Davies IG Richardson LD Stevenson L. Determinants of takeaway and fast food consumption: a narrative review. Nutr Res Rev. (2018) 31:16–34. 10.1017/S0954422417000178 29037273

[55] Duan XX Liao YT Huang JC Zhang X Dai XM Zhou J. [Associations between takeaway food nutrients and nutritional literacy of takeaway platform practitioners in Chengdu]. Zhongguo yi xue ke xue yuan xue bao Acta Academiae Medicinae Sinicae. (2021) 43:77–81. 33663667 10.3881/j.issn.1000-503X.12530

[56] Dawson SL Dash SR Jacka FN. The importance of diet and gut health to the treatment and prevention of mental disorders. Int Rev Neurobiol. (2016) 131:325–46. 10.1016/bs.irn.2016.08.009 27793225

[57] Owen L Corfe B. The role of diet and nutrition on mental health and wellbeing. Proc Nutr Soc. (2017) 76:425–6. 10.1017/S0029665117001057 28707609

